# HOW PATTERNS OF INTERGENERATIONAL SUPPORT RECEIVED PREDICT FUTURE PROVISION OF CARE? EVIDENCE FROM CHINA

**DOI:** 10.1093/geroni/igad104.1856

**Published:** 2023-12-21

**Authors:** Yinkai Zhang, Yu-Chih Chen

**Affiliations:** The University of Hong Kong, Hong Kong, Hong Kong; The University of Hong Kong, Hong Kong, Hong Kong

## Abstract

Intergenerational support refers to the exchange of financial, emotional, and physical support between generations. The types of support that older adults receive can influence their willingness to provide care in the future. However, there is limited knowledge about the different patterns of support that older adults receive. Examining these patterns can provide insight into how older adults contribute to future financial, emotional, and physical support. Using data from 5,388 older adults aged 60 and older from the China Health and Retirement Longitudinal Study, latent profile analysis was used to explore the patterns of received support. The associations between the patterns and provisions of financial, emotional, and physical support were examined using lagged OLS regression. Results showed that three patterns of received support were identified based on financial and physical aspects: financially and physically disadvantaged (low financial and physical support), engaged but financially disadvantaged (low financial but high physical support), engaged but physically moderate (high financial and medium physical support). Compared to the financially and physically disadvantaged group, those who were engaged but financially disadvantaged offer more financial and emotional support but less physical support, whereas engaged but physically moderate groups are more likely to provide emotional care. Findings suggest that policies and programs should consider the different patterns of support that older adults receive to promote more balanced support across different generations.

